# Supplementary material for: Strategies and responses to the effects of Climate Change on health systems in Sub-Saharan Africa: A scoping review protocol
Source: PLoS One. 2025 Aug 6;20(8):e0316775. doi: 10.1371/journal.pone.0316775 (PMC12327594; doi:10.1371/journal.pone.0316775)
Supplement: S1 File — (DOCX) [file pone.0316775.s001.docx]

**Table 1: Full search strategies for the electronic databases**

| **#** | **Search strings for PubMed**  **Date of search: 30/04/25**  **Time of search: 14:00Hrs** | **Results** |
| --- | --- | --- |
| 1 | "health systems" OR "healthcare systems" OR "district health systems" OR "health service systems" | 1,823 |
| 2 | 1+AND "policy strategies" OR "policy responses" OR "policy adaptation" OR "policy mitigation" OR "policy resilience" | 85 |
| 3 | 1+2+AND "Climate Change" OR "extreme weather events" OR "climate variability" OR "Global warming" | 50 |
| 4 | 1+2+3+AND (Africa OR Southern Africa OR East Africa OR Central Africa OR West Africa OR Sub-Saharan Africa OR Sub-Sahara Africa OR Sub Sahara Africa OR AND "Angola" OR "Benin" OR "Botswana" OR "Burkina Faso" OR "Burundi" OR "Cape Verde" OR "Cameroon" OR "Central African Republic" OR "Chad" OR "Comoros" OR "Democratic Republic of the Congo " OR "Republic of the Congo" OR "Cote d'Ivoire" OR "Djibouti" OR "Equatorial Guinea" OR "Eritrea" OR "Ethiopia" OR "Gabon" OR "Gambia" OR "Ghana" OR "Guinea" OR "Guinea-Bissau" OR "Kenya" OR "Lesotho" OR "Liberia" OR "Madagascar" OR "Malawi" OR "Malawi" OR "Mali" OR "Mauritania" OR "Mauritius" OR "Mozambique" OR "Namibia" OR "Niger" OR "Nigeria" OR "Rwanda" OR "Sao Tome and Principe" OR "Senegal" OR "Seychelles" OR "Sierra Leone" OR "Somalia" OR "South Africa" OR "South Sudan" OR "Sudan" OR "Swaziland" OR "Tanzania" OR "Togo" OR "Uganda" OR "Zambia" OR "Zimbabwe” | 7,810 |
|  | **MEDLINE EbscoHost**  **Date of search: 30/04/25**  **Time of search: 16:30 Hrs** |  |
| 1 | "health systems" OR "healthcare systems" OR "district health systems" OR "health service systems" | 71,285 |
| 2 | 1+ "policy strategies" OR "policy responses" OR "policy adaptation" OR "policy mitigation" OR "policy resilience" | 149 |
| 3 | 1+2+ "Climate Change" OR "extreme weather events" OR "climate variability" OR "Global warming" | 5 |
| 4 | 1+2+3+"Angola" OR "Benin" OR "Botswana" OR "Burkina Faso" OR "Burundi" OR "Cape Verde" OR "Cameroon" OR "Central African Republic" OR "Chad" OR "Comoros" OR "Democratic Republic of the Congo " OR "Republic of the Congo" OR "Cote d'Ivoire" OR "Djibouti" OR "Equatorial Guinea" OR "Eritrea" OR "Ethiopia" OR "Gabon" OR "Gambia" OR "Ghana" OR "Guinea" OR "Guinea-Bissau" OR "Kenya" OR "Lesotho" OR "Liberia" OR "Madagascar" OR "Malawi" OR "Malawi" OR "Mali" OR "Mauritania" OR "Mauritius" OR "Mozambique" OR "Namibia" OR "Niger" OR "Nigeria" OR "Rwanda" OR "Sao Tome and Principe" OR "Senegal" OR "Seychelles" OR "Sierra Leone" OR "Somalia" OR "South Africa" OR "South Sudan" OR "Sudan" OR "Swaziland" OR "Tanzania" OR "Togo" OR "Uganda" OR "Zambia" OR "Zimbabwe” | 1 |
|  | **Search strings for Scopus:**  **Date of search: 30/04/25**  **Time of search: 16:00Hrs** |  |
| 1 | "health systems" OR "healthcare systems" OR "district health systems" OR "health service systems" | 162,725 |
| 2 | 1 + "policy strategies" OR "policy responses" OR "policy adaptation" OR "policy mitigation" OR "policy resilience" | 431 |
| 3 | 1+2+ "Climate Change" OR "extreme weather events" OR "climate variability" OR "Global warming" | 17 |
| 4 | 1+2+3+"Angola" OR "Benin" OR "Botswana" OR "Burkina Faso" OR "Burundi" OR "Cape Verde" OR "Cameroon" OR "Central African Republic" OR "Chad" OR "Comoros" OR "Democratic Republic of the Congo " OR "Republic of the Congo" OR "Cote d'Ivoire" OR "Djibouti" OR "Equatorial Guinea" OR "Eritrea" OR "Ethiopia" OR "Gabon" OR "Gambia" OR "Ghana" OR "Guinea" OR "Guinea-Bissau" OR "Kenya" OR "Lesotho" OR "Liberia" OR "Madagascar" OR "Malawi" OR "Malawi" OR "Mali" OR "Mauritania" OR "Mauritius" OR "Mozambique" OR "Namibia" OR "Niger" OR "Nigeria" OR "Rwanda" OR "Sao Tome and Principe" OR "Senegal" OR "Seychelles" OR "Sierra Leone" OR "Somalia" OR "South Africa" OR "South Sudan" OR "Sudan" OR "Swaziland" OR "Tanzania" OR "Togo" OR "Uganda" OR "Zambia" OR "Zimbabwe” | 1 |
|  | **Search strings for CINAHL**  **Date of search: 30/04/25**  **Time of search: 15:00Hrs** |  |
| 1 | ( "health systems" OR "healthcare systems" OR "district health systems" OR "health service systems") | 17,673 |
| 2 | 1+ ("policy strategies" OR "policy responses" OR "policy adaptation" OR "policy mitigation" OR "policy resilience" ) | 54 |
| 3 | 1+2+("Climate Change" OR "extreme weather events" OR "climate variability" OR "Global warming" ) | 1 |
| 4 | 1+2+3+("Angola" OR "Benin" OR "Botswana" OR "Burkina Faso" OR "Burundi" OR "Cape Verde" OR "Cameroon" OR "Central African Republic" OR "Chad" OR "Comoros" OR "Democratic Republic of the Congo " OR "Republic of the Congo" OR "Cote d'Ivoire" OR "Djibouti" OR "Equatorial Guinea" OR "Eritrea" OR "Ethiopia" OR "Gabon" OR "Gambia" OR "Ghana" OR "Guinea" OR "Guinea-Bissau" OR "Kenya" OR "Lesotho" OR "Liberia" OR "Madagascar" OR "Malawi" OR "Malawi" OR "Mali" OR "Mauritania" OR "Mauritius" OR "Mozambique" OR "Namibia" OR "Niger" OR "Nigeria" OR "Rwanda" OR "Sao Tome and Principe" OR "Senegal" OR "Seychelles" OR "Sierra Leone" OR "Somalia" OR "South Africa" OR "South Sudan" OR "Sudan" OR "Swaziland" OR "Tanzania" OR "Togo" OR "Uganda" OR "Zambia" OR "Zimbabwe”) | 0 |
