# Supplementary material for: Strategies and responses to the effects of Climate Change on health systems in Sub-Saharan Africa: A scoping review protocol
Source: PLoS One. 2025 Aug 6;20(8):e0316775. doi: 10.1371/journal.pone.0316775 (PMC12327594; doi:10.1371/journal.pone.0316775)
Supplement: Attachment — Submitted filename: POINT BY POINT RESPONSE TO THE REVIEWERS.docx [file pone.0316775.s004.docx]

**POINT BY POINT RESPONSE TO THE REVIEWERS**

| **POINT** | **REVIEWER COMMENTS** | **AUTHOR’S RESPONSE** |
| --- | --- | --- |
| 1 | *Include specific numbers and relative risks regarding the prevalence/ incidence of relevant negative health outcomes in your research area, Sub-Saharan Africa (to highlight the importance).* | We thank the reviewer for suggesting this. We have added the following sentence to the introduction in paragraph 2 line No 2-3. **“It has been reported that over 40 million people were affected by floods between 2010 1nd 2020, while drought contributed to 88% of all CC related disasters in Sub-Saharan Africa”.** |
| 2 | *"heat stress" as an environmental stressor is not the only cause for premature deaths (due to CC). Air pollution, hurricanes, and floods could also play an important role? (You can clarify your sentence: "CC will cause an additional 250,000 deaths per year from malnutrition, malaria, diarrhea, and heat stress")* | We thank the reviewer for pointing this out. We have added the following sentence to the introduction first paragraph line No 4-5. “**WHO further indicates that CC environmental stressors, such as air pollution, hurricanes, and floods, will contribute to premature deaths globally.”** |
| 3 | *In your research environment, are there differences between the consequences of CC in rural and urban areas? (Perhaps this is also a relevant topic for your discussion.)* | We thank the reviewer for this recommendation. We have added the following under discussion section, first paragraph, line No 5-11. **“The health system within the region faces similar CC challenges including floods, drought and disease outbreaks and increased heat. Although these CC consequences are felt in both rural and urban areas, however, there are distinct differences between them. Climate-sensitive diseases such as waterborne diseases, malnutrition, and displacement are more pronounced in rural areas, while heat stress, air pollution, and infrastructure strain are more reported in urban areas, especially in non-informal settlements.”** |
| 4 | *Does climate change only affect the infrastructure of health systems? It may also have an impact on human resources (which are particularly lacking in Malawi) and, in the worst case, even lead to cumulative effects of climate change.* | We thank the reviewer for mentioning this. This has been addressed in the introduction, paragraph 6 from line No 1-7, and it reads. **“The effects of CC on health systems have been clarified and categorized in terms of changes in volume and patterns of health service demand from increased morbidity and mortality, reduced capacity to meet the primary and public health needs due to interruption of vital components of the health system such as infrastructure or equipment damage, increase pressure on human resource demand and interruption of other health-related services such as access to clean water, sanitation, food insecurity, disrupted energy supply and communication”.** |
| 5 | *You could describe the role of infectious disease more detalied (Malaria and the Cholera outbreak in 2022).* | We thank the reviewer for this recommendation. Brief description has been added to the introduction part, paragraph 4, line No 1-8. |
| 6 | *- Briefly describe the health care system in Sub-Saharan Africa/ Malawi and it’s challenges (less human resource, less training opportunities, limited treatment options ...) as well as the economic situation in a global context (dependency of other countries etc.) in order to understand why research is important for Sub-Saharan Africa/ Malawi. CC seems to be a major problem that adds to the structural and already existing fundamental problems.* | We thank the reviewer for the suggesting this. We have added the following sentence to the introduction part paragraph 4 line No 6-12. **“The health systems in Sub-Saharan Africa are characterized by a mix of public and private providers, with the majority being public facilities. For instance, Malawi’s health system comprises of public, private for-profit and private not-for-profit facilities, with public facilities providing free health services at the point of contact. However, the health systems in the region face similar challenges such as inadequate human resource, less training opportunities, limited treatment options as well as donor dependent due to poor funding”.** |
| 7 | *- Check consistency in terminology (e.g., climate change vs. CC).* | We thank the reviewer for noticing this. We have edited all areas. |
